# Randomized comparison of oblique and perpendicular stabilizers for minimally invasive repair of pectus excavatum

**DOI:** 10.1093/icvts/ivae040

**Published:** 2024-03-16

**Authors:** Miguel L Tedde, Rafael Lucas Costa De Carvalho, Jose Ribas Milanez De Campos, Diego Arley Gomes Da Silva, Erica Mie Okumura, Gustavo Falavigna Guilherme, Alana Cozzer Marchesi, Paulla Petrizzo, Barbara Siqueira Souto Maior, Paulo Manuel Pego-Fernandes

**Affiliations:** Divisao Cirurgia Toracica, Instituto do Coracao, Hospital das Clinicas HCFMUSP, Faculdade de Medicina, Universidade de Sao Paulo, Sao Paulo, SP, Brazil; Thoracic Surgery Division, Hospital Infantil Sabará, São Paulo, Brazil; Divisao Cirurgia Toracica, Instituto do Coracao, Hospital das Clinicas HCFMUSP, Faculdade de Medicina, Universidade de Sao Paulo, Sao Paulo, SP, Brazil; Divisao Cirurgia Toracica, Instituto do Coracao, Hospital das Clinicas HCFMUSP, Faculdade de Medicina, Universidade de Sao Paulo, Sao Paulo, SP, Brazil; Divisao Cirurgia Toracica, Instituto do Coracao, Hospital das Clinicas HCFMUSP, Faculdade de Medicina, Universidade de Sao Paulo, Sao Paulo, SP, Brazil; Divisao Cirurgia Toracica, Instituto do Coracao, Hospital das Clinicas HCFMUSP, Faculdade de Medicina, Universidade de Sao Paulo, Sao Paulo, SP, Brazil; Divisao Cirurgia Toracica, Instituto do Coracao, Hospital das Clinicas HCFMUSP, Faculdade de Medicina, Universidade de Sao Paulo, Sao Paulo, SP, Brazil; Divisao Cirurgia Toracica, Instituto do Coracao, Hospital das Clinicas HCFMUSP, Faculdade de Medicina, Universidade de Sao Paulo, Sao Paulo, SP, Brazil; Divisao Cirurgia Toracica, Instituto do Coracao, Hospital das Clinicas HCFMUSP, Faculdade de Medicina, Universidade de Sao Paulo, Sao Paulo, SP, Brazil; Divisao Cirurgia Toracica, Instituto do Coracao, Hospital das Clinicas HCFMUSP, Faculdade de Medicina, Universidade de Sao Paulo, Sao Paulo, SP, Brazil; Divisao Cirurgia Toracica, Instituto do Coracao, Hospital das Clinicas HCFMUSP, Faculdade de Medicina, Universidade de Sao Paulo, Sao Paulo, SP, Brazil

**Keywords:** Funnel chest, Chest wall, Minimally invasive surgery, Clinical trials, Protocol

## Abstract

**OBJECTIVES:**

Bar dislocation is one of the most feared complications of the minimally invasive repair of pectus excavatum.

**METHODS:**

Prospective randomized parallel-group clinical trial intending to assess whether oblique stabilizers can reduce bar displacement in comparison with regular stabilizers used in minimally invasive repair of pectus excavatum. Additionally, we evaluated pain, quality of life and other postoperative complications. Participants were randomly assigned to surgery with perpendicular (*n* = 16) or oblique stabilizers (*n* = 14) between October 2017 and September 2018 and followed for 3 years. Bar displacements were evaluated with the bar displacement index. Pain scores were evaluated through visual analogue scale and quality of life through the Pectus Excavatum Evaluation Questionnaire.

**RESULTS:**

Control group average displacement index was 17.7 (±26.7) and intervention group average displacement index was 8.2 (±10.9). There was 1 reoperation in each group that required correction with 2 bars. Bar displacement was similar among groups (*P* = 0.12). No other complications were recorded. There was no statistically significant difference on pain score. There was a significant difference between pre- and postoperative composite scores of the participants’ body image domain and psycho-social aspects in both groups. The difference between the pre- and postoperative participants’ perception of physical difficulties was greater and statistically significant in the intervention group.

**CONCLUSIONS:**

There was no statistical difference in the use of perpendicular or oblique stabilizers, but the availability of different models of stabilizers during the study suggested that this can be advantageous. The trial is registered at ClinicalTrials.gov, number NCT03087734.

**Figure:**
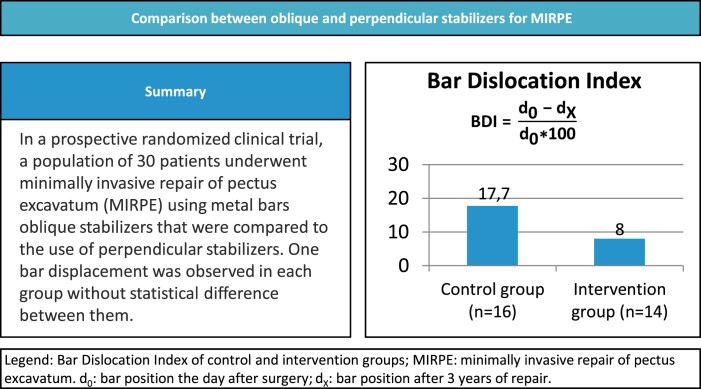

## INTRODUCTION

Pectus excavatum (PE) is associated with shortness of breath, exercise intolerance, low self-esteem and depression [1–3] and to date there is no effective non-invasive treatment [4]. The minimally invasive repair of pectus excavatum (MIRPE) described by Nuss has become the standard of care [5] and is associated with a significant improvement in performing physical activities [6, 7] and in quality of life (QoL) regarding body image and psycho-social aspects [8, 9].

Among the complications, bar displacement stands out as the most serious one due to aesthetic impairment and possible need for reoperation [10]. Placing stabilizers perpendicular to the metal bar is the most used way in the attempt to keep it in proper position. Even so, it is estimated that up to 7% of MIRPE procedures present bar displacement [11].

The point is that these stabilizers only are stable when they are perpendicular to the ribs and it only occurs if they are placed in a medial position. This causes the hinge points to also be medial, reducing the range of chest wall that is being corrected [12].

With the intention of reducing this complication, we considered the hypothesis that stabilizers positioned obliquely to the bar would remain in a perpendicular position to the ribs, even when positioned more lateral on thoracic wall, thus providing greater stability to the set.

**Figure 1:**
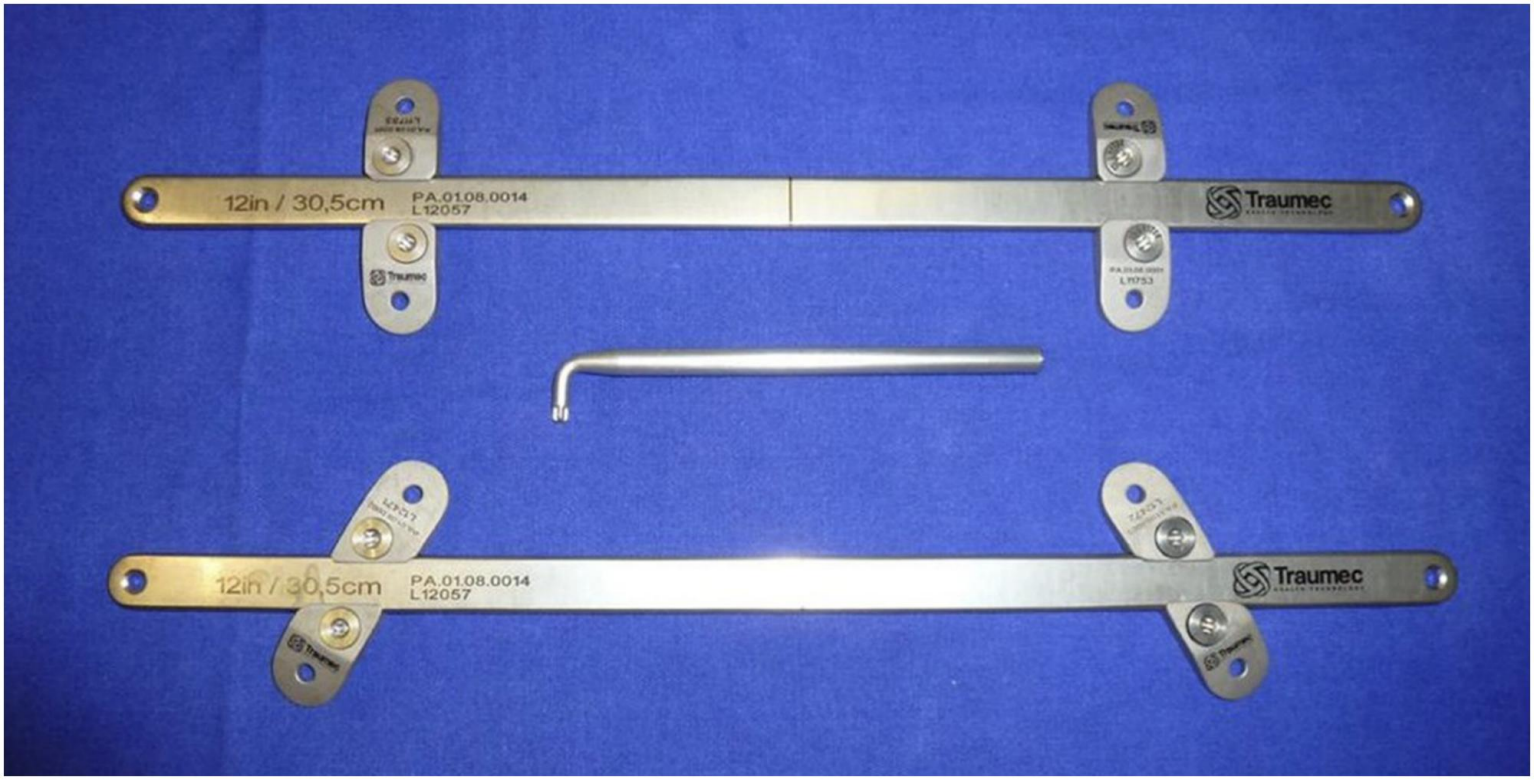
Set of metallic bar and stabilizers developed for the study.

It was developed a new set of bars, in addition to perpendicular and oblique stabilizers (Traumec Tecnologia e Implantes, Rio Claro, Brazil). Other characteristics were added: smooth bars eliminating the notches at their ends, larger channel for the stabilizers and narrower bridges between its halves; built in pressure screws and titanium instead of steel intending to avoid high blood metal levels induced by steel bars (Fig. 1) [13, 14].

A randomized trial was designed intending to test the hypothesis that an oblique stabilizer would adapt better to the ribs and that could reduce the chance of dislocation of the bar in comparison to perpendicular stabilizers. The results of the study according to the Consolidated Standards of Reporting Trials (CONSORT) are presented here [15].

## PATIENTS AND METHODS

### Ethics statement

The study was approved by the University of São Paulo Medical School IRB (CAPPesq) number 1.633.063 on 12 July 2016. A formal written consent was obtained from all participants, and if child participants, the formal consent was obtained from their parent/guardians. The trial is registered at ClinicalTrials.gov, number NCT03087734.

### Study design and population

This is a prospective parallel-group surgical trial with balanced randomization (1:1) in which 32 participants were recruited to undergo MIRPE in a study conducted in Brazil. As there was no precise data in the literature indicating which types of PE needed to be corrected with more than 1 metal bar, the surgeon would decide during surgery if there was a need to place a 2nd bar for aesthetic or safety reasons.

Eligible participants were individuals with PE aged 11 or over. Exclusion criteria were participants with congenital heart diseases, complex chest wall anomalies, previous failed PE repair, previous thoracic operations, bleeding disorders or major anaesthetic risks.

The study took place at the Department of Thoracic Surgery of the Heart Institute (InCor) from Hospital das Clinicas da Universidade de Sao Paulo. MIRPE procedures were performed between October 2017 and March 2019 and follow-up was completed after bar removal, in September 2022.

### Clinical information

Demographic data were collected preoperatively and PE severity measurements determined by chest computerized tomography. Thirty-two patients were randomly assigned to be operated with the perpendicular stabilizers (control group) or the oblique stabilizer (intervention group). All MIRPE were done following the thoracoscopic technique described in the literature [16–18].

Patients received epidural anaesthesia and analgesia in the early postoperative period, as well as opioid and conventional analgesics after discharge. They were followed with outpatient visits on the 15th postoperative day, 1 month, 3 months, 6 months and annually for a minimum period of 3 years after surgery, when the bars were removed [19].

### Outcomes

The primary end-point was to compare the incidence of bar displacement in the control (perpendicular) and intervention (oblique) groups through the bar displacement index (BDI). The BDI is a ratio between bar position in the early postoperative period (d0) and at the end of the analysed period (dX), which is calculated with the formula: BDI = d0 – dX/d0 × 100. This measurement was obtained from postoperative lateral chest radiographs as the distance from the posterior superior end of the sternal body to the upper border of the metal bar [20].

The secondary end-points were to assess the incidence of pre- and postoperative complications such as: vascular, lung, diaphragm or cardiac injury; seroma, haematoma, wound infection or dehiscence, pleural or pericardial effusion, pneumothorax, haemothorax, atelectasis, pneumonia, bar rotation, subcutaneous emphysema, allergy, bone erosion and recurrence, through clinical follow-up.

The other secondary end-points were to evaluate postoperative pain intensity through visual analogue scale [21] and QoL through the application of the Pectus Excavatum Evaluation Questionnaire (PEEQ) to patients and their parents preoperatively and 6 months after surgery.

The PEEQ is a disease-specific QoL evaluation tool developed at Old Dominion University where the patient is addressed with 11 questions and the parents with 13, with answers given following a Likert-type scale from 1 to 4. These questions were grouped into 3 domains (body image, psycho-social aspects and physical difficulties) for patients and 4 domains (psycho-social aspects, physical difficulties, self-awareness and parental concern) were assessed for parents [22].

Sample size calculation was hampered by study’s funding source limitations and was only able to afford 32 surgical procedures, defining the sample. For allocation, a computer-generated list of random numbers was used. Participants were randomly assigned following simple randomization procedures to 1 of 2 treatment groups. The allocation sequence was concealed from the researchers enrolling participants in sequentially numbered and opaque envelopes. Corresponding envelopes were opened just before surgery.

### Statistical analysis

Data analysis was presented descriptively by means and standard deviations, and by absolute and relative frequency. To compare differences between groups, the non-parametric Mann–Whitney *U*-test or the parametric independent *t*-test was used. The analyses were performed using the Statistical Package for Social Sciences 23.0 computer software with a significance level of 5% for statistical tests.

## RESULTS

**Figure 2:**
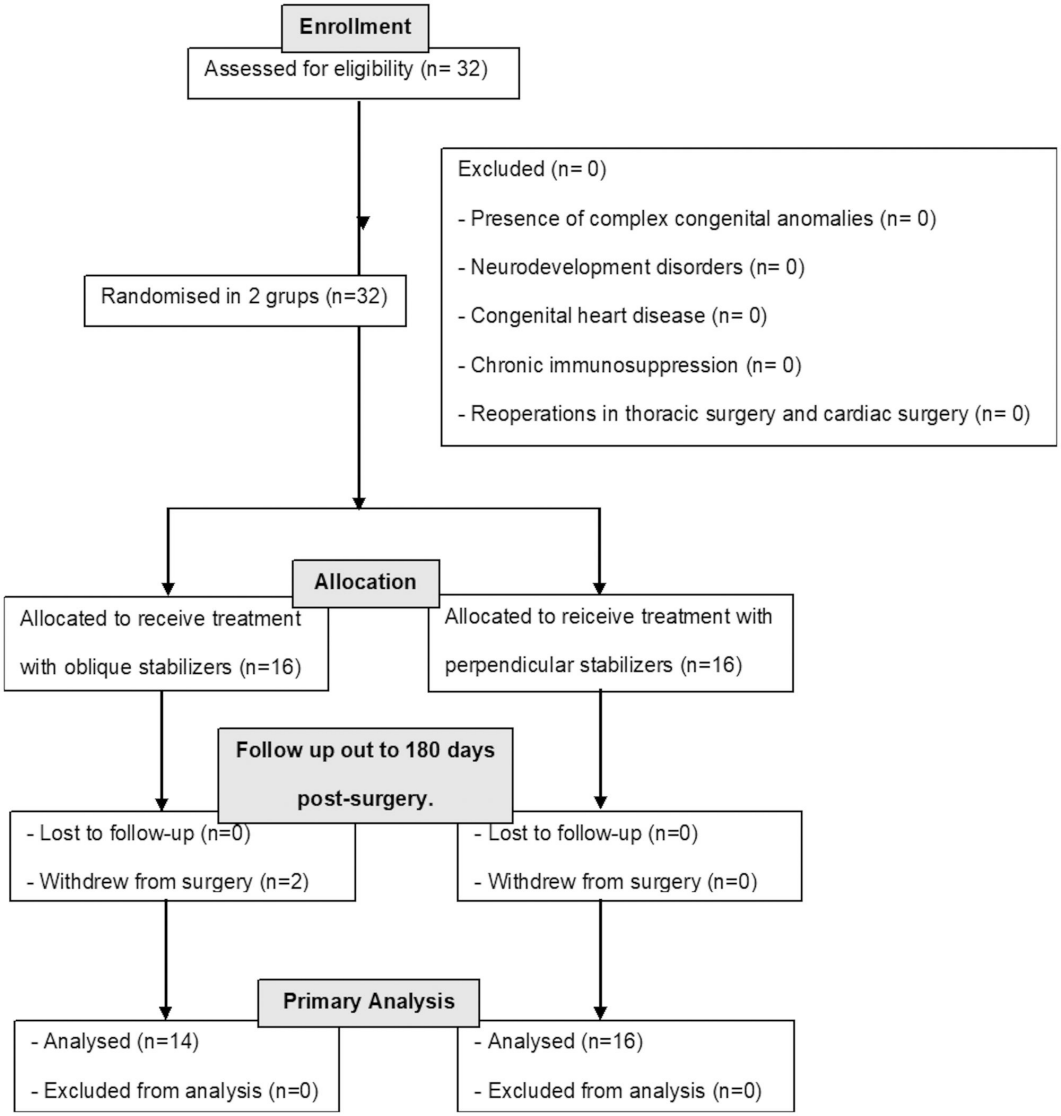
CONSORT enrolment and follow-up diagram.

Thirty-two patients were recruited. The random distribution resulted in 2 groups of 16 participants each, to be treated with perpendicular stabilizers (control group) and or with oblique stabilizers (intervention group). Two patients from the intervention group withdrew from undergoing surgery (Fig. 2).

The groups were homogeneous in terms of demographic characteristics. Recruitment began in January 2017 and the participants were submitted to MIRPE between October 2017 and September 2018. They were followed-up until the removal of the bars, 3 years after surgery.

Mean age was 17 ± 3.3 (range 14–27) years, with male predominance (90%). The main demographic and perioperative characteristics are described in Table 1.

**Table 1:** Demographic, surgical characteristics and length of stay of groups

Variable	Control group (*n* = 16)	Intervention group (*n* = 14)	*P*-value
Male	14 (87.5%)	13 (92.9%)	0.62
Age	17 (±2.92)	17.64 (±3.54)	0.75
Weight (kg)	59.25 (47.25–61.97)	58.28 (±9.57)	0.72
Height (cm)	1.72 (±0.06)	1.77 (±0.09)	0.11
Body mass index	19.53 (±3.45)	18.51 (±2.47)	0.46
Haller index	4.33 (±1.22)	3.93 (±0.83)	0.50
Operated with 2 bars	2 (12.5%)	1 (7.1%)	0.62
Reoperation	1 (6.3%)	1 (7.1%)	0.92
Length of stay	5 (4–6)	4.50 (4–6)	0.42

Parametric: Independent samples *t*-test (mean ± standard deviation); nonparametric: Mann–Whitney *U*-test (median, interquartile range); nominal: chi-square test.

Analysis was conducted with all operated participants. Two individuals from the control group and 1 from the intervention group underwent MIRPE with 2 bars instead of 1 due to intraoperative decision.

There was displacement of the bar in both groups, according to the BDI assessment. As shown in Table 2, control group had an average BDI of 17.7 (±26.7), while the intervention group had an average BDI of 8.2 (±10.9). Although the control group had a higher mean BDI value than the oblique stabilizers group, this difference was not statistically significant (*P* = 0.12).

**Table 2:** Bar dislocation index and postoperative pain scores of control and intervention groups

Variable	Control group (*n* = 16)	Intervention group (*n* = 14)	*P*-value
Bar dislocation index	17.7 (±26.7)	8.2 (±10.9)	0.12
Analogue visual scale of pain			
PO1	8 (7–9)	7 (5–9)	0.608
PO2	7 (4.25–8)	7 (5.75–9)	0.423
PO3	7 (4.25–8)	7 (5–8)	0.759
PO4	4.93 (±3.08)	5 (±2.98)	0.518
PO5	3.75 (±2.84)	5 (±3.08)	0.850
Hospital discharge	3.50 (± 2.89)	4.78 (±3.06)	0.808
PO15	3.50 (0.25–5.75)	3.5 (0–7.25)	0.667
PO30	1 (0–4)	0 (0–4.25)	0.697
PO60	1 (0–3.5)	0 (0–3.25)	0.637
PO180	0 (0–2)	0 (0–0)	0.070

Parametric variables were presented as mean and standard deviation (SD). Non-parametric variables were presented as median and interquartile range. Difference between the means (p) of the groups was analysed using the *T*-test Independent samples for parametric variables and Mann–Whitney *U*-test for non-parametric variables.

PO: postoperative day.

**Figure 3:**
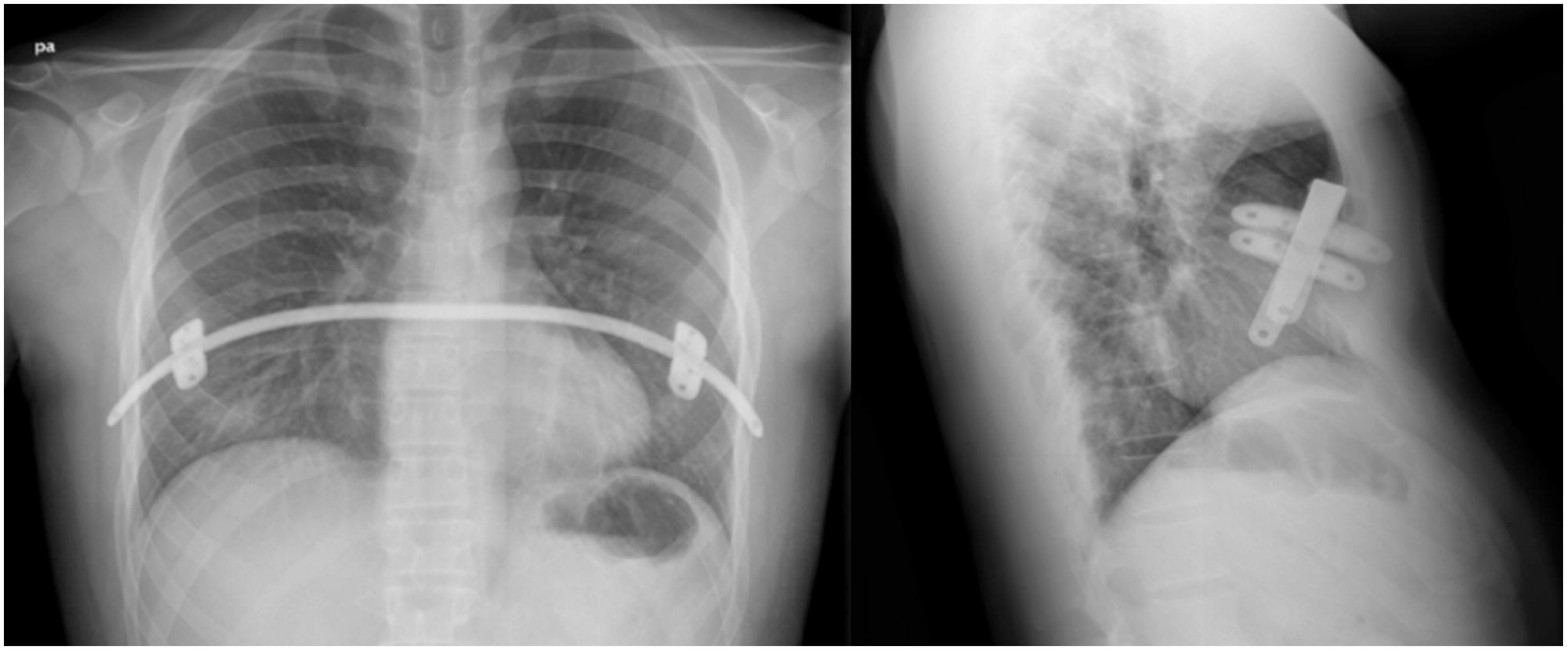
Bar displacement in a patient from control group.

**Figure 4:**
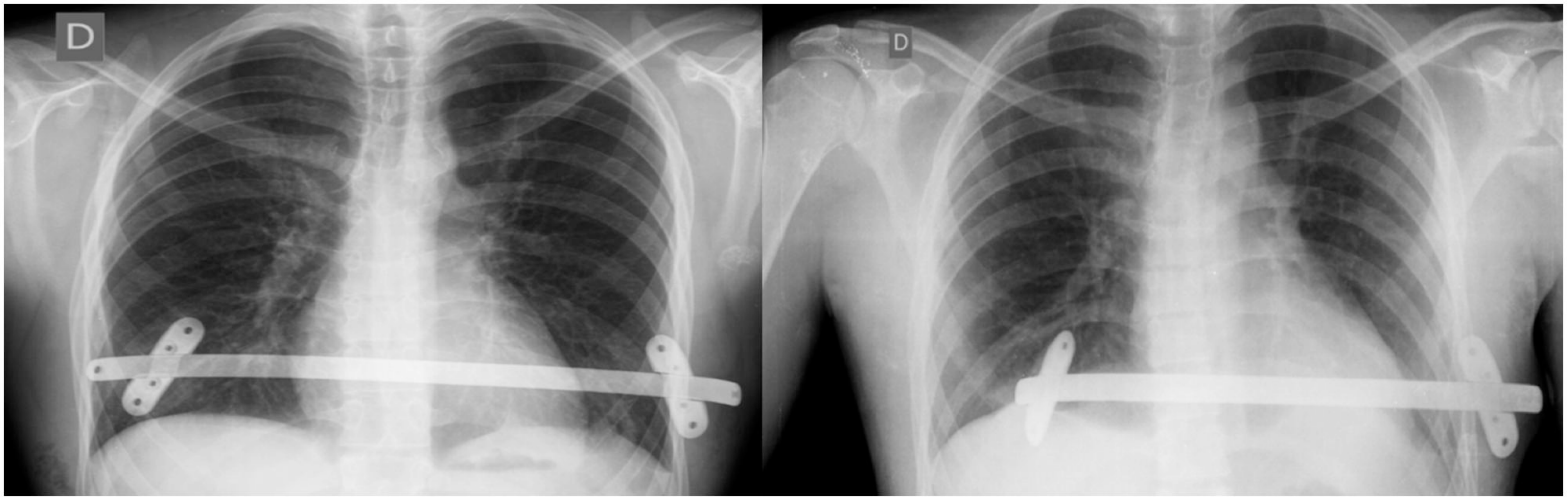
Bar displacement in a patient from intervention group.

There was 1 case of bar dislocation due to bar flipping on its own axis in the control group (Fig. 3) and 1 case in the intervention group with a different mechanism: the bar ripped the intercostal muscle and slipped into the thoracic cavity (Fig. 4). Both cases required reoperation and they were corrected with 2 bars each with uneventful recovery. In addition to these cases, no other complications were recorded. It is worth mentioning that the patients who needed reoperation due to this displacement had BDI rates of 42.8 (intervention group) and 121.9 (control group), much higher than the average.

### Pain scores

Pain scores marked on the visual analogue scale reveal severe pain in the 1st postoperative days considering both groups, even using patient-controlled epidural analgesia during hospitalization. This score reduces over time and most patients do not report pain after 30 days of the procedure. When comparing the 2 groups, considering the same postoperative period, there was no statistically significant difference in terms of pain score (Table 2).

### Quality of life assessment

There was a significant improvement in the postoperative QoL after 6 months of the operation. The difference observed between pre- and postoperative composite scores of the participants’ body image domain and psycho-social aspects in both control and intervention arms was significant. The difference between the pre- and postoperative scores on participants’ perception of physical difficulties was greater, and statistically significant, in the intervention group than in the control group [23]. It was also observed a significant improvement of parent’s perception of the participants’ QoL before and after MIRPE in both groups.

When comparing the questionnaire responses of the 2 groups, there was no statistical difference for any of the questions. The detailed results of the study regarding QoL assessment of the same groups of patients using PEEQ 6 months after MIRPE were reported elsewhere [23].

Although there were no established parameters for evaluating the characteristics of the new developed material, some observed aspects seem to have the potential to positively impact the surgical outcome of patients undergoing MIRPE.

The fact that the stabilizers had wider channels facilitated the introduction of metallic bars even if they were already moulded. The pressure screws incorporated in the structure of the stabilizers prevent from detachment to become foreign bodies and favour a practical fixation of the stabilizers at any point of the bars. It has also been noted that upon removal, smooth bars appear to show less peribar calcification than bars with grooved ends. This makes sense if considered that the bars were made with serrated ends precisely to promote scar tissue formation [24].

## DISCUSSION

### Limitations

One limitation of the study is the small number of participants included, which was determined by budget restriction. Another point that contributed to weakening the treatment standardization is the fact that there are no clear parameters to indicate when a PE should be corrected with 1 or more bars. Some papers suggest that it is advisable to use 2 bars for MIRPE to decrease the risk of displacement in asymmetrical or deep defects [25, 26]. It ends up being dependent on the intraoperative decision of the surgeon, if considered inadequate the correction with only 1 bar, for safety or aesthetic reasons.

Two bars were used in 3 upfront cases (2 in control and 1 in the intervention group) and in both cases when reoperation was needed. Although an intention to treat analysis was conducted and the proposed type of stabilizers in each group were respected, there is a potential risk of bias due to better stabilization of the chest wall when two bars were used.

The new set of bars and stabilizers was used in a diverse group of patients in terms of age, severity and sex, with complication rates similar to those reported in literature [27]. The three-year follow-up reinforces the impression that the set is safe and effective. In this series, except for bar displacement, no other complications were observed.

Bar dislodgement is described as the most common relevant complication, with an incidence of 9.2%, resulting in recurrence of the defect and impairing patient satisfaction [28]. Furthermore, in rarer events, it can add severity when there is compression of mediastinal structures or organ injury, cases in which reoperation is required [20].

It is shown that stabilizers, whether or not attached with suture to the ribs or musculature, are capable of reducing bar dislocation incidence from 15 to 5%. But this does not solve the problem [24].

To test the hypothesis that oblique stabilizers could reduce the displacement of the bars, and as this model of stabilizer was not available on the market, the solution found was to manufacture it. The rationale was that a stabilizer that has an oblique orientation in relation to the bar ends up in a perpendicular position to the ribs, increasing the contact area and possibly increasing the stability of the set.

The difference in BDI between the control and the intervention group was not statistically significant, possibly due to the small sample size. It was observed that the measure was useful for estimating smaller displacements but not so adequate as an objective assessment of indication for reoperation as it was suggested when it was created.

In the series, there was 1 case that required reoperation due to bar displacement in each group, and these patients who required corrective surgery had a much higher BDI than the cut-off value of 8.7 defined in the publication [20], significantly increasing mean BDI of both groups. It seems that the common clinical criteria (recurrence of the defect associated with radiological evidence of bar displacement) should remain the best parameter to indicate a reoperation.

Regarding bar displacement, it is important to highlight that these events had different mechanisms. While in the control group, the displacement was a typical bar rotation, in the intervention group, it occurred because the oblique stabilizer slipped into the chest after the participant had suddenly raised the arm, which must have increased the intercostal space [22, 25].

**Figure 5:**
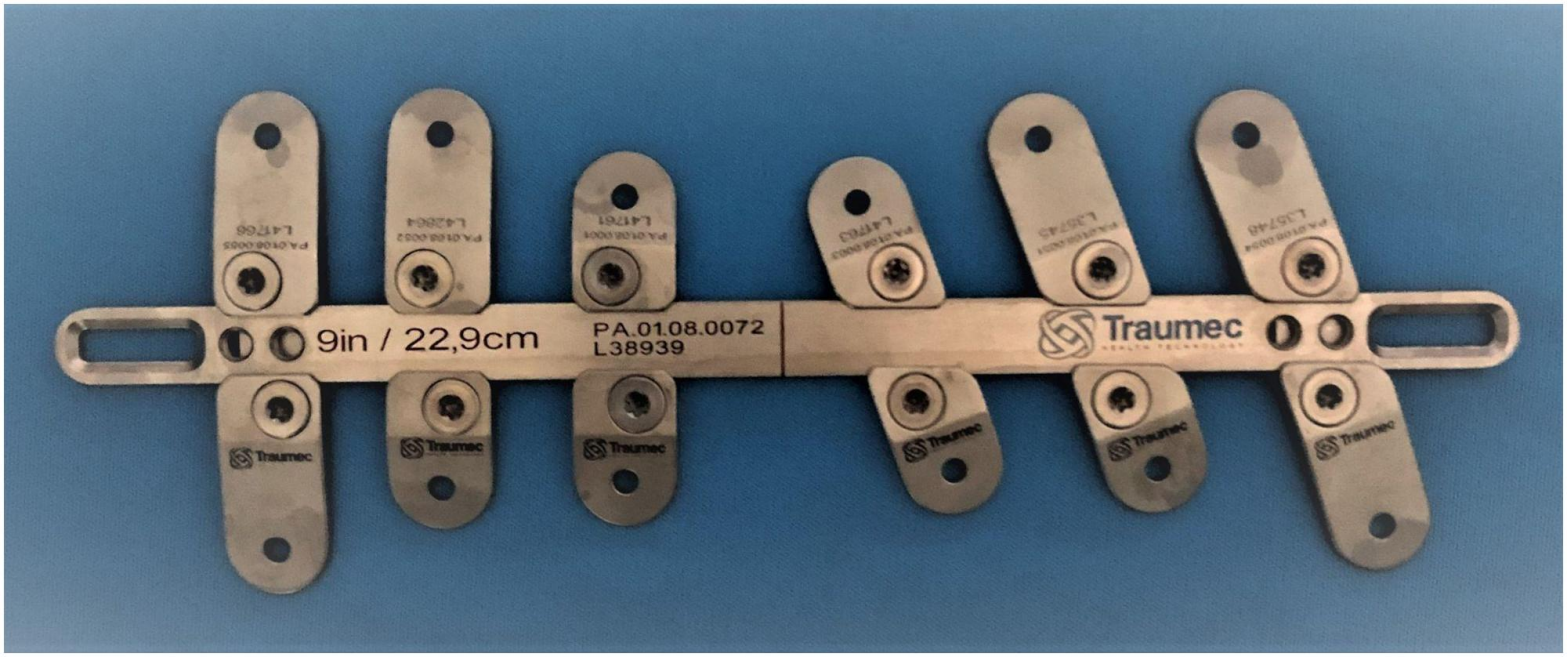
The longer, asymmetric and standard options of perpendicular and oblique stabilizers.

This event triggered the hypothesis that stabilizers with longer ends would avoid these complications. Although not used because they were not part of the trial design, the stabilizers manufactured as final products now come in 3 different sizes: standard, asymmetrical and longer (Fig. 5). This will allow the surgeon to choose during surgery which model best fits the patient’s anatomy.

Although there was no significant difference between the control and the intervention group, the findings of our study regarding pain are in agreement with the literature, which shows decreasing and tolerable character with the use of epidural anaesthesia, with most patients reporting no significant pain after 30 days of the procedure [29].

Likewise, we have seen great improvement in QoL life after surgery both in the control and intervention group [23]. The decremental pattern of pain associated with great improvements in QoL positively favours the procedure as the main resource in repair of PE.

## CONCLUSION

In the tested sample, no statistically significant difference was found between the use of the perpendicular or oblique stabilizer in relation to bar displacement, pain or quality of life. The subjective impression of the developed material suggests that there are advantages in using smooth bars, stabilizers with fixation screws, and different sizes and shapes. New studies are needed to confirm these subjective observations.

## Data Availability

The data underlying this article will be shared on reasonable request to the corresponding author.

## ABBREVIATIONS

BDI: Bar displacement index
CONSORT: Consolidated Standards of Reporting Trials
MIRPE: Minimally invasive repair of pectus excavatum
PE: Pectus excavatum
QoL: Quality of life
